# Supplementary material for: A miniature CRISPR–Cas10 enzyme confers immunity by inhibitory signalling
Source: Nature. Author manuscript; Available in PMC 2025 Dec 24. (PMC12657230; doi:10.1038/s41586-025-09569-9)
Supplement: Supplementary Material [file NIHMS2117476-supplement-Supplementary_Material.pdf]

A miniature CRISPR-Cas10 enzyme confers immunity by inhibitory signaling

Erin E. Doherty[1,2,3*], Benjamin A. Adler[1,2,3*], Peter H. Yoon[1,2], Kendall Hsieh[2], Kenneth Loi[2], Emily G. Armbruster[4], Arushi Lahiri[2], Cydni S. Bolling[2], Xander E. Wilcox[5], Amogha Akkati[6], Anthony T. Iavarone[3], Joe Pogliano[4], Jennifer A. Doudna[1-3,7-10†]

[1]Innovative Genomics Institute, University of California, Berkeley, California 94720, USA;

[2]Department of Molecular and Cell Biology, University of California, Berkeley, California 94720, USA;

[3]California Institute for Quantitative Biosciences (QB3), University of California, Berkeley, California 94720, USA;

[4]School of Biological Sciences, University of California San Diego, La Jolla, California 92093, USA;

[5]Department of Microbiology and Immunology, Cornell University, Ithaca, New York 14853, USA;

[6]Department of Plant and Microbial Biology, University of California, Berkeley, California 94720, USA;

[7]Gladstone Institutes, University of California, San Francisco, California 94114, USA;

[8]Howard Hughes Medical Institute, University of California, Berkeley, California 94720, USA;
[9]Department of Chemistry, University of California, Berkeley, California, USA;

[10]MBIB Division, Lawrence Berkeley National Laboratory, Berkeley, California 94720, USA;

Supplementary Figures

Supplementary Fig. 1. TLC and gel Source data.
Supplementary Fig. 2. Sequence logo from an alignment of 163 mCpol representatives from PF18182.
Supplementary Fig. 3. Topological analysis between 2TMβ Proteins from Panoptes and CBASS systems.
Supplementary Fig. 4. Conservation of β-barrel domain nucleotide binding residues.

Supplementary File 1. Plasmid information.
Supplementary File 2. Phage information.

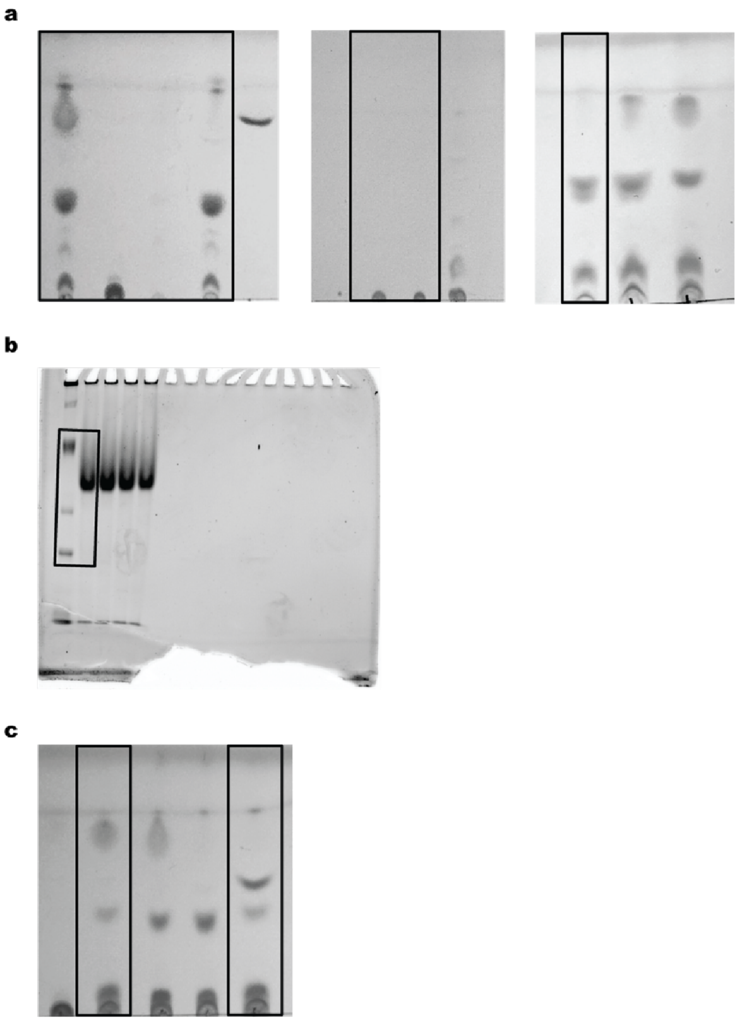

Supplementary Fig. 1. TLC and gel source data. Boxed area shows region represented in the main text or supplementary information. (a) Source TLC images from Fig. 1a. (b) Source gel image from Extended Data Fig. 5d. (c) Source TLC images from Extended Data Fig 6 d.

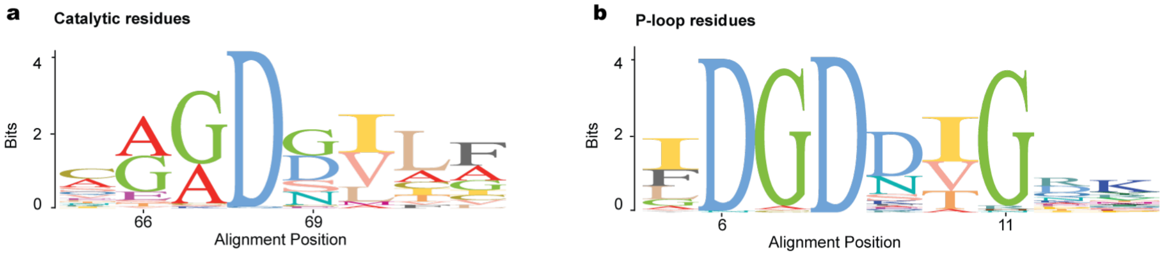

Supplementary Fig. 2. Sequence logo from an alignment of 163 mCpol representatives from PF18182. (a) Semi-conserved predicted “catalytic residues” based on sequence motif GGDD common to NTases (AADG in ECOR31 mCpol, where Alignment Position 68 corresponds with D57). (b) “P-loop” or phosphate binding loop residues which are common to ATP- and GTPbinding enzymes. Sequence logo alignment position corresponds to residues 5-12 in ECOR31 mCpol. The P-loop consensus sequence GXXXXGK(T/S) where the consensus sequence starts at position 7 and appears less conserved in the last two residues.

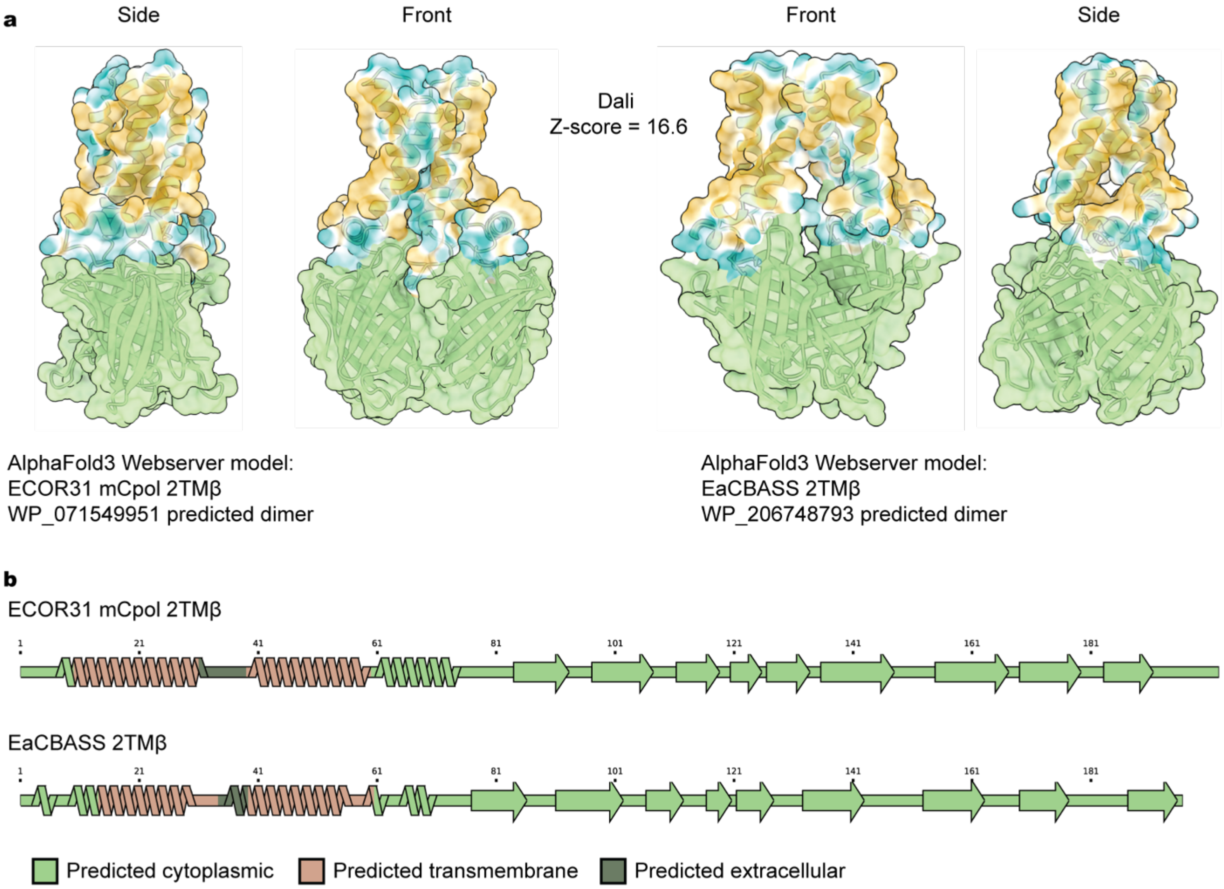

- **Supplementary Fig. 3. Topological analysis between 2TMβ Proteins from Panoptes and CBASS systems.** (a) Comparison of predicted Alphafold3 (PMID: 38718835) dimers for ECOR31-derived 2TMβ (OptE) (left) and characterized EaCBASS-derived 2TMβ (Cap15) (PMID: 34784509) (right). Predicted dimers are presented with front and side views with predicted Dali Z-score (PMID: 35610055). Conserved putative transmembrane regions are colored by hydrophobicity, where tan is more hydrophobic and teal is more hydrophilic. Conserved β-barrel region is shown in green. (b) Transmembrane prediction using TMHMM (PMID: 11152613) shows conserved topologies between Panoptes- and CBASS-derived 2TMβ proteins. Predicted transmembrane regions are colored in tan, while predicted cytosolic and extracellular regions are colored in light and dark green respectively. Secondary structure representation of 2TMβ is derived from predicted Alphafold3 dimers (Chain A) and plotted using ssDraw (PMID: 37786684) with TMHMM-derived colors.

```
                   130        140        150        160        170        180
Consensus    XXVEXXLKIX XXXXKIXMHG XTXXSXTXSX TXXXXXXEXD XXXLXYXYKX XPKTPSXXXT
ECOR31 TM2β  FEVRAKIKQA LLVTKIEMHG PTVKSVTLEA TPT---KELD NNKLYYVYKS TPKNPS---W
EaCap15      TKVEFPLEIK ADFFSIKMKG NTTIGRTYSN YCKVVRAEDD SFELVYMFKV FNDTPSITDT
YaCap15      NTWEGELKIV QTWDKVRIHL KTKASHSDSV TASIIYDKGI GYQLLYNYRN QPKTGEEHLT

                   190        200        210        220        230
Consensus    SXYXGXAXFR VIDX-XXXXX XGXYXTXR-- --GXXTXGXI XIXRIX---- -----XX
ECOR31 TM2β  SEYIGSTIFD VIESNNALQL SGRYYTDR-- ----KSVGRI SIKRISLNTD SDISFY*
EaCap15      SFYEGAARLR VIDI-KTMNM KGVFWTNRCW ENGKNTAGII ELSK------ -----EV
YaCap15      S-HVGFAEFR -FDA-DLKSA EGHYFNGQ-- --GRATYGTM TITRIE---- -----HA
```

Supplementary Fig. 4. Conservation of β-barrel domain nucleotide binding residues. Alignment of a segment of the β-barrell nucleotide binding domain of *Escherichia albertii* Cap15 and *Yersinia* Cap 15 with 2TMβ from ECOR31 Panoptes. Highlighted residues (corresponding to T129, Y153, Y155, Y188 and M200 in *Ea*Cap15) were found to be functionally important for cyclic dinucleotide binding by Duncan-Lowey et al (PMID: 34784509) .
