# Supplementary material for: A miniature CRISPR–Cas10 enzyme confers immunity by inhibitory signalling
Source: Nature. 2025 Oct 1;647(8091):997–1004. doi: 10.1038/s41586-025-09569-9 (PMC12657230; doi:10.1038/s41586-025-09569-9)
Supplement: Supplementary file 2 [file 41586_2025_9569_MOESM2_ESM.pdf]

Corresponding author(s): Doudna, Jennifer A.

Last updated by author(s): 8/15/2025

Reporting Summary

Nature Portfolio wishes to improve the reproducibility of the work that we publish. This form provides structure for consistency and transparency in reporting. For further information on Nature Portfolio policies, see our Editorial Policies and the Editorial Policy Checklist.

Statistics

For all statistical analyses, confirm that the following items are present in the figure legend, table legend, main text, or Methods section.

n/a	Confirmed	
☐	☒	The exact sample size (*n*) for each experimental group/condition, given as a discrete number and unit of measurement
☐	☒	A statement on whether measurements were taken from distinct samples or whether the same sample was measured repeatedly
☒	☐	The statistical test(s) used AND whether they are one- or two-sided<br>*Only common tests should be described solely by name; describe more complex techniques in the Methods section.*
☒	☐	A description of all covariates tested
☒	☐	A description of any assumptions or corrections, such as tests of normality and adjustment for multiple comparisons
☐	☒	A full description of the statistical parameters including central tendency (e.g. means) or other basic estimates (e.g. regression coefficient) AND variation (e.g. standard deviation) or associated estimates of uncertainty (e.g. confidence intervals)
☒	☐	For null hypothesis testing, the test statistic (e.g. *F*, *t*, *r*) with confidence intervals, effect sizes, degrees of freedom and *P* value noted<br>*Give P values as exact values whenever suitable.*
☒	☐	For Bayesian analysis, information on the choice of priors and Markov chain Monte Carlo settings
☒	☐	For hierarchical and complex designs, identification of the appropriate level for tests and full reporting of outcomes
☒	☐	Estimates of effect sizes (e.g. Cohen's *d*, Pearson's *r*), indicating how they were calculated

Our web collection on statistics for biologists contains articles on many of the points above.

Software and code

Policy information about availability of computer code

Data collection	Mass spectrometry data acquisition was controlled using Xcalibur software version 2.0.7. X-ray data collection was controlled using the BLU-ICE graphical Interface. Size Exclusion Chromatography data acquisition was controlled using the UNICORN 7.3 software from Cytiva. Fluorescence microcoscopy images were collected using using DeltaVision SoftWoRx version 6.5.2.
Data analysis	The following software was used: GraphPad Prism 10.2.2, MAFFT v7.490 in Geneious Prime v2023.0.1., Python 3.7.3., Adobe Photoshop (21.2.0) and Adobe Illustrator (24.2). Growth curve and CRISPRi-ART visualizations were performed using the Seaborn v 0.13.2and matplotlib v3.7.2 package in Python. Crystallographic data were processed with the SSRL autoxds script with an I/sigI cutoff ≥ 1.50. Molecular replacement and structural refinement were carried out in PHENIX version 1.21.2-5419. The model was built and adjusted using COOT version 0.9.8.95 EL. Structures were visualized in ChimeraX-1.7.1. Mass spectrometry raw data were converted to mzXML format using msconvert 3.0.19052.1 from the Galaxy platform and data were processed using the open source software MZmine 3.9.0. DefenseFinder Web Service v2.0.0 was used for defense association analysis. Custom Python scripts used for sequence mining, genomic neighborhood analysis, and taxonomic analysis will be available in Zenodo upon publication.

For manuscripts utilizing custom algorithms or software that are central to the research but not yet described in published literature, software must be made available to editors and reviewers. We strongly encourage code deposition in a community repository (e.g. GitHub). See the Nature Portfolio guidelines for submitting code & software for further information.

Data

Policy information about availability of data

All manuscripts must include a data availability statement. This statement should provide the following information, where applicable:

- Accession codes, unique identifiers, or web links for publicly available datasets
- A description of any restrictions on data availability
- For clinical datasets or third party data, please ensure that the statement adheres to our policy

The structure of mCpol-ApNHpp has been deposited under PDB ID: 9NWN and is publicly available. Structures accessed in this work are publicly available under PDB ID: 6IFK, 6IFL and 3W2W. Mass spectrometry data has been deposited at Figshare project number: 261113. Fluorescence microscopy images have been deposited at Figshare (project number: 260219). Plasmids and plasmid sequences are available on Addgene under the article title (Deposition #86474).

Research involving human participants, their data, or biological material

Policy information about studies with human participants or human data. See also policy information about sex, gender (identity/presentation), and sexual orientation and race, ethnicity and racism.

Reporting on sex and gender	N/A
Reporting on race, ethnicity, or other socially relevant groupings	N/A
Population characteristics	N/A
Recruitment	N/A
Ethics oversight	N/A

Note that full information on the approval of the study protocol must also be provided in the manuscript.

Field-specific reporting

Please select the one below that is the best fit for your research. If you are not sure, read the appropriate sections before making your selection.

☒ Life sciences ☐ Behavioural & social sciences ☐ Ecological, evolutionary & environmental sciences

For a reference copy of the document with all sections, see nature.com/documents/nr-reporting-summary-flat.pdf

Life sciences study design

All studies must disclose on these points even when the disclosure is negative.

Sample size	No statistical methods were used to predetermine sample size. Sample sizes were chosen to balance feasibility with information content, while including repetitions to verify reproducibility. We performed experiments in biological triplicate (n = 3) to facilitate measurements of mean and dispersion unless otherwise noted.
Data exclusions	No data were excluded from the analyses.
Replication	Unless specified otherwise, all reported data represent the average of three biological replicates. All attempts at replication were successful.
Randomization	This study did not perform experiments that require sample randomization as this study does not involve allocation into different experimental groups.
Blinding	This study did not perform experiments that require blinding since experimental outcomes are objective, quantitative readouts obtained directly from instruments (e.g. growth curves) or using predefined parameters (e.g. plaque quantification).

Reporting for specific materials, systems and methods

We require information from authors about some types of materials, experimental systems and methods used in many studies. Here, indicate whether each material, system or method listed is relevant to your study. If you are not sure if a list item applies to your research, read the appropriate section before selecting a response.

Materials & experimental systems

n/a	Involved in the study
☒	☐ Antibodies
☒	☐ Eukaryotic cell lines
☒	☐ Palaeontology and archaeology
☒	☐ Animals and other organisms
☒	☐ Clinical data
☒	☐ Dual use research of concern
☒	☐ Plants

Methods

n/a	Involved in the study
☒	☐ ChIP-seq
☒	☐ Flow cytometry
☒	☐ MRI-based neuroimaging

Plants

Seed stocks	*Report on the source of all seed stocks or other plant material used. If applicable, state the seed stock centre and catalogue number. If plant specimens were collected from the field, describe the collection location, date and sampling procedures.*
Novel plant genotypes	*Describe the methods by which all novel plant genotypes were produced. This includes those generated by transgenic approaches, gene editing, chemical/radiation-based mutagenesis and hybridization. For transgenic lines, describe the transformation method, the number of independent lines analyzed and the generation upon which experiments were performed. For gene-edited lines, describe the editor used, the endogenous sequence targeted for editing, the targeting guide RNA sequence (if applicable) and how the editor was applied.*
Authentication	*Describe any authentication procedures for each seed stock used or novel genotype generated. Describe any experiments used to assess the effect of a mutation and, where applicable, how potential secondary effects (e.g. second site T-DNA insertions, mosiacism, off-target gene editing) were examined.*
